# CircSNHG5 Sponges Mir-495-3p and Modulates CITED2 to Protect Cartilage Endplate From Degradation

**DOI:** 10.3389/fcell.2021.668715

**Published:** 2021-07-01

**Authors:** Jian Zhang, Shen Hu, Rui Ding, Jinghong Yuan, Jingyu Jia, Tianlong Wu, Xigao Cheng

**Affiliations:** ^1^Department of Orthopedics, The Second Affiliated Hospital of Nanchang University, Nanchang, China; ^2^Institute of Orthopedics of Jiangxi Province, Nanchang, China; ^3^Institute of Minimally Invasive Orthopedics, Nanchang University, Nanchang, China

**Keywords:** intervertebral disc degeneration, cartilage endplate, competing endogenous RNA, circular RNA, microRNA

## Abstract

**Background:**

Intervertebral disc degeneration (IDD) is a highly prevalent degenerating disease that produces tremendous amount of low back and neck pain. The cartilage endplate (CEP) is vitally important to intervertebral discs in both physiological and pathological conditions. In addition, circular RNAs (circRNAs) have been shown to be involved in the regulation of various diseases, including IDD. However, the particular role of circRNAs in cervical vertebral CEP degeneration remains unclear. Here, we examined the unique role of circRNAs in CEP of patients with cervical fracture and degenerative cervical myelopathy (DCM).

**Methods:**

Human competitive endogenous RNA (ceRNA) microarray was performed by previous research. Western blot (WB), immunofluorescence (IF), quantitative RT-PCR (qRT-PCR), luciferase assay, and fluorescence *in situ* hybridization (FISH) were employed to analyze the function of circSNHG5 and its downstream effectors, miR-495-3p, and CITED2.

**Results:**

We demonstrated that circSNHG5 expression was substantially low in degenerative CEP tissues. Knockdown of circSNHG5 in chondrocytes resulted in a loss of cell proliferation and followed by degradation of extracellular matrix (ECM). In addition, circSNHG5 was shown to sponge miR-495-3p and modulate the expression of the downstream gene CITED2. This mechanism of action was further validated *via* overexpression and knockdown of CITED2.

**Conclusion:**

Our findings identified a novel circSNHG5-miR-495-3p axis responsible for IDD progression. Future investigations into IDD therapy may benefit from targeting this axis.

## Introduction

Low back and neck pain are widespread global phenomena that lead to disability (Boogaarts and Bartels, 2015; Hartvigsen et al., 2018). One of the main causative factors of these pains is intervertebral disc degeneration (IDD; Samartzis et al., 2011; Gibson et al., 2018). The intervertebral disc is a largely avascular tissue that receives nutrition from capillary diffusion at the cartilage endplate (CEP) situated above and below the intervertebral disc (Gullbrand et al., 2014; Malandrino et al., 2014). Prior studies on IDD have examined nucleus pulposus (NP) extensively, but little work has been done regarding the involvement of CEP. The CEP is an integral part of intervertebral disc and is composed of hyaline cartilage and extracellular matrix (ECM; Wang et al., 2012; Newell et al., 2017). Destruction of CEP can result in ECM nutrient deprivation and can promote IDD induction and development (Grunhagen et al., 2006). Although the involvement of CEP destruction in IDD is well established, the underlying mechanisms remain unknown. Therefore, investigating CEP relevant pathway(s) is urgent and necessary for the advancement of high-efficacy IDD therapy.

Circular RNA (circRNA) belongs to endogenous non-coding RNAs (Chen, 2016; Chen and Schuman, 2016). They are reported to have enhanced stability due to the closed loop structure and participate in a variety of physiological and pathological pathways (Kristensen et al., 2019). Among the many functions of circRNAs, their function as competing endogenous RNAs (ceRNAs) has gained increasing popularity in recent years. As ceRNAs, circRNA can bind to miRNA and serves as a sponge of microRNA (miRNA) to modulate downstream gene expression (Hansen et al., 2013; Zheng et al., 2016). As such, circRNAs were found to be involved in the development of numerous diseases, including heart failure, cancer, and even neurological diseases (Devaux et al., 2017; Dube et al., 2019; Li and Han, 2019). Interestingly, circRNAs are also thought to be involved in the pathogenesis of IDD (Li et al., 2019). For instance, Xiao et al. (2020) demonstrated that circRNA_0058097 sequesters miR-365a-5p and modulates HDAC4 expression in its regulation of CEP degeneration. On the other hand, Song et al. (2018) revealed elevated circRNA_104670 levels in human IDD tissues, which sponges miRNA-17-3p and regulates expression of MMP-2. However, the expression profiles and potential functions of circRNAs in the cervical vertebral CEP remains to be examined.

Here, we analyzed the circRNA expression profile in degenerative CEP tissues versus healthy CEP tissues and discovered that circSNHG5 (has_circ_0077254) was markedly downregulated in degenerative CEP. Next, we demonstrated that circSNHG5 serves as a miR-495-3p sponge and modulates downstream genes to regulate IDD progression. The conclusions from this study will offer new insight into the contribution of circRNA in degenerative CEP and provide potential molecular targets for IDD therapy.

## Materials and Methods

### Ethics Statement

This study received approval from the Institutional Ethical Review Board of Nanchang University and obtained written informed agreements from all patients before use of their tissues in the study. CEP samples were retrieved from patients undergoing surgery at the Second Affiliated Hospital of Nanchang University.

### Clinical Specimens

Human degenerative CEP samples were sourced from 21 surgical patients with degenerative cervical myelopathy (DCM) undergoing discectomy. For control, CEP samples were retrieved from 21 patients undergoing decompressive surgery to correct cervical spinal trauma-related neuronal defects. Magnetic resonance imaging (MRI) was performed in all patients prior to the surgery. According to the method described by a previous study (Rajasekaran et al., 2008), we distinguished two groups of endplate tissues based on the preoperative MRI. CEP tissues were surgically extracted, quick frozen in liquid nitrogen, and kept at −80°C until further analysis. All data involving the patients and the harvested samples are presented in Supplementary Table 1.

### Cell Culture

The human chondrocytic cell line C28/I2 was maintained in Dulbecco’s modified Eagle medium (DMEM/F12; Thermo Fisher Scientific, China) containing 10% fetal bovine serum (FBS; Thermo Fisher Scientific, China) and 1% penicillin–streptomycin (Invitrogen) at 37°C and 5% CO_2_.

### Cell Transfection

CircSNHG5 small interfering RNAs (siRNAs) were purchased from GenePharma (Shanghai, China); miR-495-3p inhibitor and mimics were obtained from RiboBio (Guangzhou, China), and pcDNA3.1 vector for CITED2 came from Genechem (Shanghai, China). Transient transfections were conducted with Lipofectamine 3000 reagent (Invitrogen), following manufacturer’s guidelines. All sequences mentioned above are summarized in Supplementary Table 2.

### Quantitative Real-Time PCR

Total RNA was isolated from cells using Trizol reagent (Invitrogen, United States). PrimeScript RT Master Mix (Takara, Japan), SYBR Premix Ex Taq II kits (Takara, Japan), and RT detection system ABI7500 (Applied Biosystems, United States) were employed in the assessment of circRNA, mRNA, and GAPDH gene expression, following the manufacturer’s guidelines. The bulge-Loop miRNA quantitative RT-PCR (qRT-PCR) Starter Kit (RiboBio, Guangzhou, China) was used to quantify miRNA and U6 gene expression. Each experiment was repeated 3×. GADPH was used as an endogenous control for circRNA and gene transcripts, whereas U6 was used as an endogenous control for miRNA. We confirmed the head-to-tail splicing of the circRNA *via* Sanger sequencing. All primers used in this study are listed in Supplementary Table 2.

### Cell Viability Assay

Cell viability was evaluated using CCK8 assay. Cells were seeded into 96-well plates at a density of 2,000 cells per well and then cultured for 0, 12, 24, 48, and 72 h, before addition of the CCK-8 solution (CCK-8, TransGen Biotech), followed by incubation at 37°C for 2 h before recording optical density (OD) values at 450 nm.

### Western Blotting and Immunofluorescence Analysis

The total protein from chondrocytes or CEP tissue was extracted using radioimmunoprecipitation assay (RIPA) lysis buffer (Beyotime, China). Protein quantification was performed with the BCA Protein Quantitation Kit (Beyotime, China). Next, the protein was separated on an SDS-polyacrylamide gel before transferring into a PVDF membrane (Millipore, United States). The membrane was then blocked with skim milk, incubated overnight at 4°C with primary antibody, washed 3×, and incubated at room temperature for 2 h with secondary antibody. The antibodies used in this study were collagen type II (1:3,000; Abcam, United Kingdom), aggrecan (1:1,000; Abcam, United Kingdom), MMP-13 (1:3,000; Abcam, United Kingdom), CITED2 (1:500; Santa Cruz Biotechnology), GAPDH (1: 5,000; Abcam, United Kingdom), and goat anti-rabbit secondary antibody (1:5,000; Abcam, United Kingdom). The protein bands were quantified using Image J software and GADPH was used as a loading control.

### Target Prediction of ceRNA

Possible circSNHG5 interaction with target-specific miRNAs was screened using the circRNA interactome website.^1^ Furthermore, TargetScanHuman7.2^2^ and miRDB^3^ were employed for the identification of potential select miRNA targets.

### Luciferase Reporter Assay

Both wild-type (WT) and mutated (MUT) forms of circSNHG5 and CITED2 3′UTR were inserted into dual luciferase reporter plasmids by RiboBio (Guangzhou, China). These plasmids were incorporated into 293T cells, along with miR-495-3p mimics or negative control (NC) using Lipofectamine 3000 (Invitrogen, United States). Forty-eight hours following incubation, the luciferase activity was measured using the Dual-Luciferase Assay System (Promega, United States).

### RNA Fluorescence *in situ* Hybridization

We purchased Alexa Fluor^®^ 488-labeled circSNHG5 probe and Cy3-labeled miR-495-3p probe from Servicebio (Wuhan, China). Slices were fixed with 4% paraformaldehyde for 20 min, permeated with 0.5% triton X-100 for 10 min, PBS-washed 3× for a total of 15 min, exposed to drops of pre-hybridization solution at 37°C for 1 h, introduced to hybridization solution with Alexa Fluor^®^ 488-labeled circSNHG5 probe, or Cy3-labeled miR-495-3p probe overnight at 37°C, combined with circSNHG5 and miR-495-3p, liquid- and phosphate-washed at 37°C with continuous mixing, exposed to DAPI for 8 min in the dark, and, lastly, imaged with a Nikon laser scanning confocal microscope (Nikon Instruments, Japan). The images were recorded in the probe sequence summarized in Supplementary Table 2.

### Statistical Analysis

Our findings are presented here as average ± standard deviation (SD). The SPSS 25.0 (IBM, United States) software was employed for statistical analysis, which further employed one-way ANOVA for multiple comparisons, or Student’s *t*-test for simple one-on-one comparisons. *P* < 0.05 (two-sided) was statistically significant.

## Results

### Comparing circRNA Gene Expression Patterns in Degenerating CEP Tissues, Relative to Healthy CEP

**FIGURE 1:**
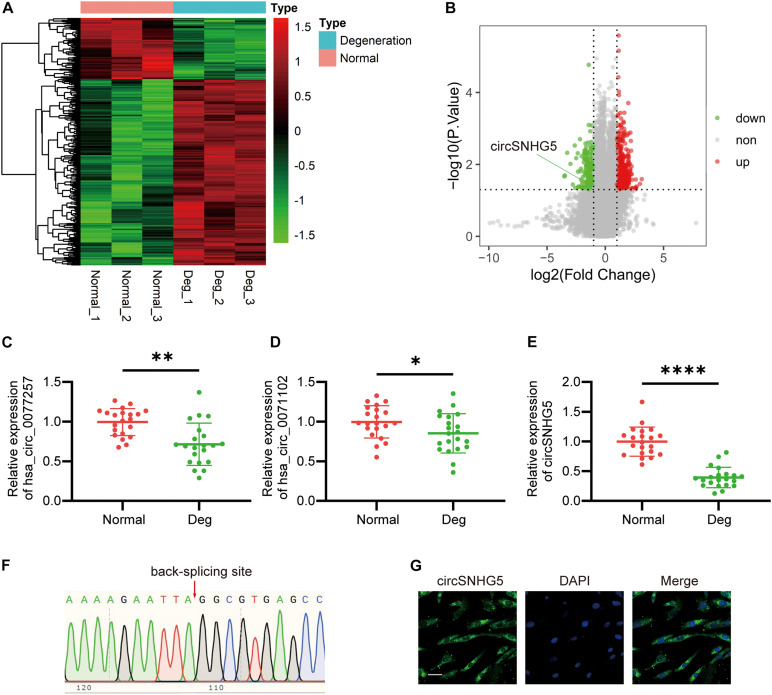
circSNHG5 expression is downregulated in degenerative cartilage endplate (CEP) tissues and predominantly localized in the cytoplasm. **(A)** Heat map of differentially expressed circRNAs with fold changes (absolute value) > 2 and *P*-value < 0.05 between the two groups. **(B)** Volcano plots show circRNAs differentially expressed; upregulated in red, downregulated in green. **(C–E)** The expression of hsa_circ_0077257, hsa_circ_0071102, and circSNHG5 in different CEP tissues of 21 patients and 21 control samples (^∗^*P* < 0.05, ^∗∗^*P* < 0.01, and ^∗∗∗^*P* < 0.001). **(F)** Sanger sequencing confirmed the back-splice junction sites of circSNHG5. **(G)** RNA FISH showed that circSNHG5 was predominantly localized in the cytoplasm. circSNHG5 probes were labeled with Alexa fluor 488. The nuclei were stained with DAPI. Scale bar, 20 μm.

We have conducted a prior study where human competitive endogenous RNA (ceRNA) microarray was performed to compare degenerative CEP to healthy CEP (Yuan et al., 2020). All information resulting from that microarray can be found in the gene expression omnibus (GEO) database^4^ under the accession no. GSE153761. The fold change value (FC) ≥ 2.0 or logFC ≥ 1.0, *P*-value < 0.05 as the threshold for differential screening to identify differential genes. Using hierarchical clustering, we mapped out circRNA expression profile in both degenerative and normal CEP samples (Figure 1A). A volcano plot was completed to illustrate the differential circRNA expression in these tissues (Figure 1B). We demonstrated 578 circRNAs that were differentially regulated in degenerative CEP samples, as compared to healthy tissues. Among them, 435 circRNAs were highly expressed, whereas the expression of 143 was markedly suppressed. Furthermore, among the top 30 downregulated circRNAs, we selected three circRNAs with the highest expression values for further analysis.

### CircSNHG5 Expression Is Downregulated in Degenerative CEP Tissues and Is Predominantly Localized in the Cytoplasm

To further confirm the human ceRNA microarray data, we detected the levels of hsa_circ_0077257, hsa_circ_0071102, and circSNHG5 in degenerative and healthy CEP tissues, using qRT-PCR. Similar to the microarray data, all three circRNAs exhibited marked suppression in degenerative CEP tissues, as opposed to healthy CEP, with circSNHG5 displaying the largest decrease (Figures 1C–E). Using Sanger sequencing, we next confirmed a back-splice junction of circSNHG5 (Figure 1F). Relevant data on circSNHG5 are summarized in Supplementary Table 2. Lastly, using RNA FISH, we demonstrated that the circSNHG5 is primarily found in the cytoplasm (Figure 1G). Based on these results, we determined the localization of circSNHG5, and it is heavily downregulated in degenerative CEP tissues.

### CircSNHG5 Suppression Drives ECM Degeneration and Reduces Chondrocyte Proliferation

**FIGURE 2:**
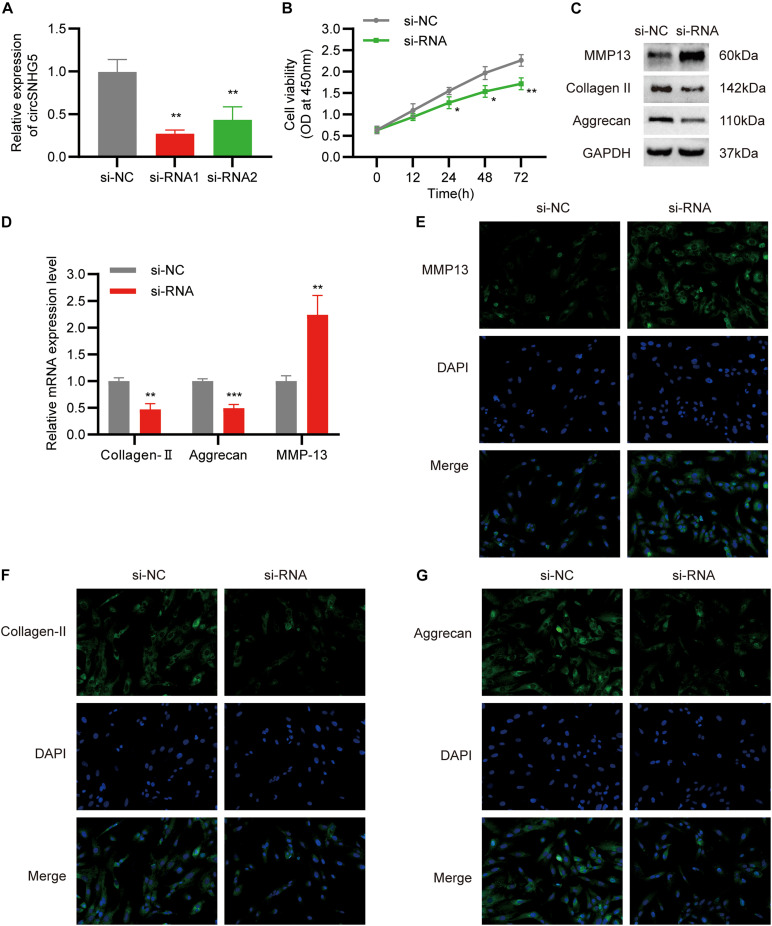
Silencing circSNHG5 leads to functional depletion. **(A)** Expression analyses of circSNHG5 knockdown efficiency by two different siRNAs in chondrocytes (**P < 0.01). **(B)** The effect of circSNHG5 on cell proliferation was determined by CCK8 assay (*P < 0.05, **P < 0.01). **(C)** Western blot analysis of MMP13, Collagen II, and Aggrecan when circSNHG5 was downregulated. The optical density analysis was performed from the results of three independent experiments of Western blot samples. **(D)** Relative mRNA expression test by qRT-PCR (**P < 0.01, ***P < 0.001). **(E–G)** Immunofluorescence (IF) staining for MMP13, Collagen II, and Aggrecan after transfection with siRNA. Scale bar, 40 μm.

To elucidate the role of circSNHG5 in CEP chondrocytes, two circSNHG5-specific siRNAs were used to knockdown circSNHG5 expression in C28/I2 cells (Figure 2A). Among them, siRNA1 was deemed as the most efficient and was used in subsequent experiments. C28/I2 cells were transiently transfected with circSNHG5-specific siRNA and assessed for changes in cell proliferation. As shown in Figure 2B, circSNHG5 knockdown dramatically reduced cell proliferation, as evidenced by CCK-8 assay. Moreover, the protein levels of MMP13 increased, whereas Collagen II and Aggrecan decreased in circSNHG5-silenced C28/I2, as opposed to negative control (Figures 2C,E–G). These changes were also evident at the transcript level, as shown by RT-qPCR (Figure 2D). Taken together, circSNHG5 silencing ceased chondrocytic cell proliferation and stimulated ECM degradation.

### CircSNHG5 Sponges miR-495-3p to Suppress Its Expression

**FIGURE 3:**
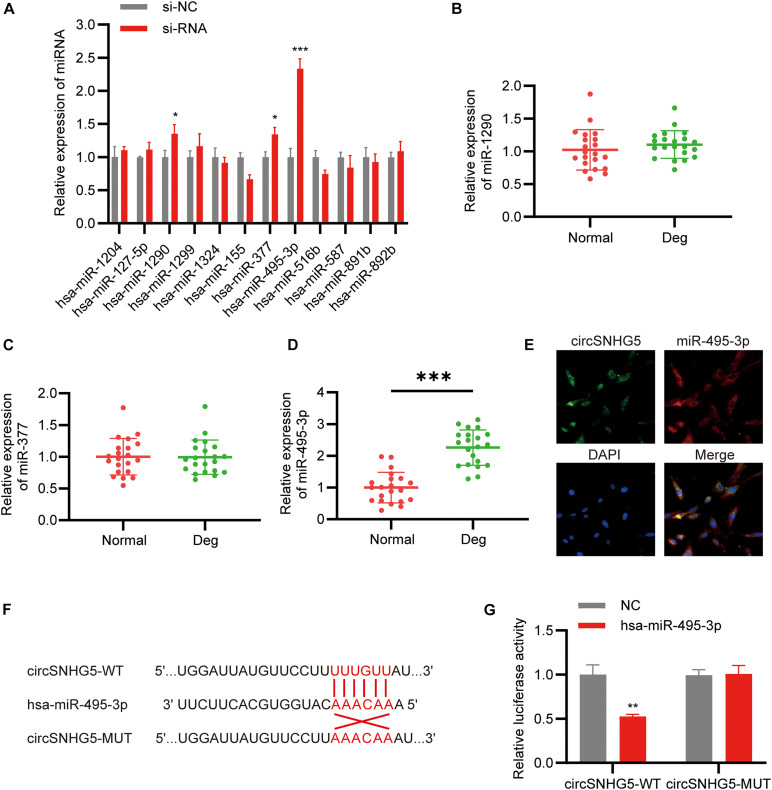
CircSNHG5 serves as a sponge for the miR-495-3p to inhibit its expression. **(A)** The expression of predicted miRNAs in chondrocytes after transfection with siRNA (*P < 0.05, ***P < 0.001). **(B–D)** qRT-PCR analysis confirmed the upregulated miRNAs in different CEP tissues of 21 patients and 21 control samples (***P < 0.001). **(E)** RNA FISH detection of the subcellular localizations of circSNHG5 and miR-495-3p. Both molecules were co-localized and both were cytoplasmic. miR-495-3p probes were labeled with Cy3, whereas circSNHG5 probes were tagged with Alexa fluor 488. Scale bar, 20 μm. **(F)** The binding region of miR-495-3p in circSNHG5 3′UTR is shown. **(G)** Luciferase reporter analysis of either wild-type or mutant circSNHG5 3′-UTR activity. miR-495-3p was co-transfected with the wild-type or mutant vector. The presented values are the mean ± SEM of three different preparations (**P < 0.01).

CircSNHG5 is a cytoplasmic circRNA, where it enforces its post-transcriptional control. The Circular RNA Interactome^5^ was employed to predict the target miRNAs of circSNHG5. A total of 12 miRNAs were predicted to combine with the circSNHG5. Using qRT-PCR, we analyzed alterations in the 12 miRNAs after circSNHG5 silencing in C28/I2 cells. Based on our results, miR-495-3p, miR-377, and miR-1290 expression was markedly elevated upon circSNHG5 silencing (Figure 3A). Assessing the levels of these miRNAs in degenerative CEP tissues, we found miR-495-3p expression to be the most elevated, relative to healthy CEP (Figures 3B–D). Using FISH assay, we further demonstrated that circSNHG5 and miR-583 co-localize in the cytoplasm (Figure 3E). Lastly, using luciferase reporter assays, we revealed that Luc-circSNHG5 (a reporter containing the complete circSNHG5 sequence in frame with the 3′-untranslated region of luciferase) activity was strongly suppressed in miR-495-3p incorporated chondrocytes (Figure 3F). Alternately, luciferase activity of Luc-circSNHG5-MUT (containing altered miR-495-3p binding sites) remained unchanged (Figure 3G). In all, these data suggest that circSNHG5 directly interacts with miR-495-3p and regulated its expression in chondrocytes.

### Silencing miR-495-3p Rescues the Functional Depletion Caused by circSNHG5 Knockdown

**FIGURE 4:**
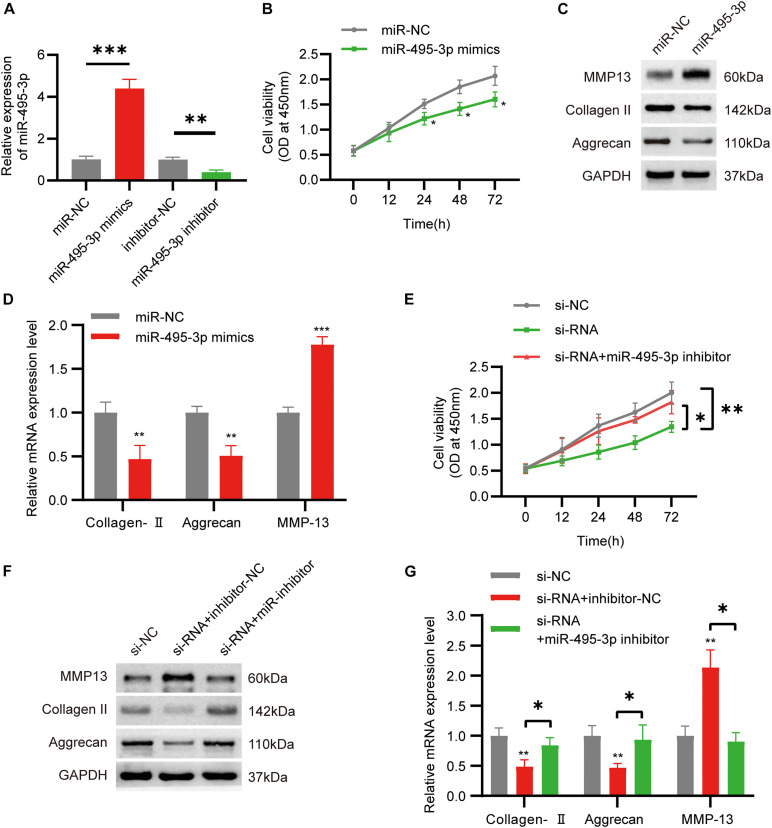
Function of miR-495-3p in chondrocytes and miR-495-3p inhibitor reversed the effects of siRNA. **(A)** The expression of miR-495-3p was assessed by qRT-PCR and transfected with miR-495-3p mimics and miR-495-3p inhibitor (^∗∗^*P* < 0.01, ^∗∗∗^*P* < 0.001). **(B)** The effect of miR-495-3p overexpression on cell proliferation *in vitro* was determined by CCK8 assay (^∗^*P* < 0.05). **(C)** Western blot analysis of MMP13, Collagen II, and Aggrecan transfected with miR-495-3p mimics. The optical density analysis was performed from the results of three independent experiments of Western blot samples (^∗^*P* < 0.05, ^∗∗^*P* < 0.01). **(D)** Relative mRNA expression test by qRT-PCR (^∗∗^*P* < 0.01, ^∗∗∗^*P* < 0.001). **(E)** Chondrocytes were co-transfected with a miR-495-3p inhibitor and siRNA; cell proliferation was monitored by CCK-8 assay (^∗^*P* < 0.05, ^∗∗^*P* < 0.01). **(F)** Western blot analysis of MMP13, Collagen II, and Aggrecan co-transfected with a miR-495-3p inhibitor and siRNA. The optical density analysis was performed from the results of three independent experiments of Western blot samples. **(G)** Relative mRNA expression test by qRT-PCR (^∗^*P* < 0.05).

To examine the role of miR-495-3p in chondrocytes, we either overexpressed or knocked down miR-495-3p in chondrocytes (Figure 4A). Based on our results, miR-495-3p incorporation into C28/I2 cells strongly suppressed cell proliferation (Figure 4B), reduced Collagen II and Aggrecan expression, and augmented MMP13 expression, relative to negative control (Figures 4C,D). Notably, miR-495-3p silenced rescued function depletion of circSNHG5 knockdown (Figures 4E–G). In brief, miR-495-3p depletion restored the effects of circSNHG5 knockdown.

### MiR-495-3p Directly Interacts With CITED2 to Suppress Cell Proliferation and Promote ECM Degeneration

**FIGURE 5:**
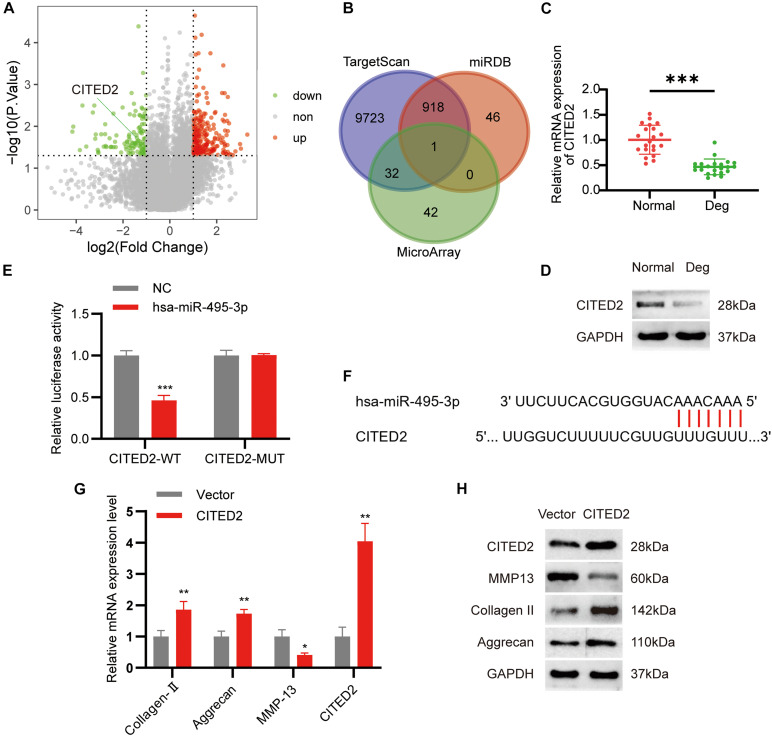
MiR-495-3p targets the CITED2 and CITED2 promotes cell proliferation inhibit ECM degeneration. **(A)** Volcano plot of the significantly upregulated and downregulated mRNAs. **(B)** Venn diagram demonstrating the intersection of downregulated mRNAs and predicted target mRNAs. **(C,D)** The expression levels of CITED2 in CEP tissues were measured in 21 patients and 21 controls by qRT-PCR and Western blot (***P < 0.001). **(E)** Luciferase reporter analysis of either wild-type or mutant CITED2 3′-UTR activity. miR-495-3p was co-transfected with the wild-type or mutant vector. The presented values are the mean ± SEM of three different preparations (***P < 0.001). **(F)** 3′-UTR region of CITED2 mRNA was found to harbor a binding site for miR-495-3p. **(G,H)** The expression of CITED2, MMP13, Collagen II, and Aggrecan expression levels was assessed by qRT-PCR and Western blotting (*P < 0.05, **P < 0.01).

To further examine the downstream effects of the circSNHG5/miR-495-3p axis, we screened the mRNA expression dataset from our microarray analysis for differentially expressed genes (DEGs). We found 171 highly expressed and 75 low-expression transcripts in the degenerative CEPs, relative to healthy CEP controls. The DEGs are illustrated as a volcano plot in Figure 5A. Next, we employed TargetScan and miRDB databases to predict potential downstream target genes for miR-495-3p. According to the Venn diagram, CITED2 was selected by both databases as a possible target for miR-495-3p (Figure 5B). Similar to the microarray data, we demonstrated both CITED2 transcript and protein expression to be markedly low in degenerative CEP tissues (Figures 5C,D). Subsequently, we transiently co-transfected HEK293T cells with luciferase reporter plasmids containing WT or MUT CITED2 sequences and miR-495-3p mimics or empty vectors. We revealed that miR-495-3p incorporated cells increased WT CITED2 luciferase activity, whereas MUT CITED2 activity remained unchanged, thereby suggesting that miR-495-3p interacts with CITED2 to regulate its activity (Figures 5E,F). Moreover, CITED2-overexpressing cells demonstrated low MMP13 levels and high Collagen II and Aggrecan expression (Figures 5G,H), which supports healthy ECM. Based on these results, endogenous CITED2 expression can be closely associated to IDD pathogenesis.

### Overexpression of CITED2 Regains Both circSNHG5 Inhibition- and the miR-495-3p Overexpression-Mediated Lost Functions in Chondrocytes

**FIGURE 6:**
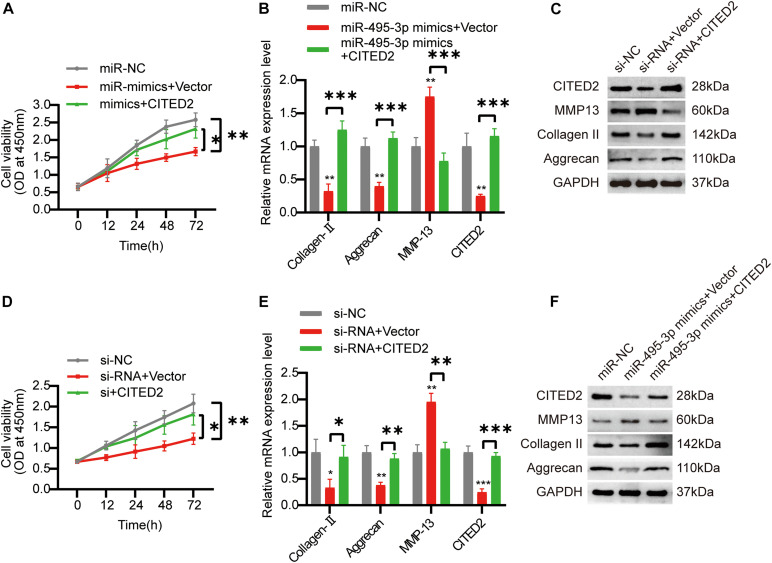
Overexpression of COL5A1 rescues the functional depletion caused by silencing circSNHG5. **(A)** The effect of CITED2 overexpression on miR-495-3p knockdown-mediated cell proliferation was measured by CCK-8 assay (*P < 0.05, **P < 0.01). **(B,C)** CITED2, MMP13, Collagen II, and Aggrecan expression levels were analyzed by qRT-PCR and Western blotting. The presented values are the means ± SD of three different preparations (***P < 0.001). **(D)** The effect of CITED2 overexpression on circSNHG5 knockdown-mediated cell proliferation was measured by CCK-8 assay (*P < 0.05, **P < 0.01). **(E,F)** CITED2, MMP13, Collagen II, and Aggrecan expression levels were analyzed by qRT-PCR and Western blotting. The presented values are the means ± SD of three different preparations (*P < 0.05; **P < 0.01; ***P < 0.001).

To confirm the circSNHG5/miR-495-3p/CITED2 axis, we next overexpressed CITED2 in C28/I2 cells. We revealed that CITED2-overexpressing cells rescued the low cell viability seen with miR-495-3p-overexpressed and circSNHG5-silenced cells (Figures 6A,D). Moreover, using Western Blotting (WB) and qRT-PCR, we showed that CITED2 incorporation can abrogate the effects of circSNHG5 knockdown or miR-495-3p mimic on Collagen II and Aggrecan (Figures 5B,C,E,F). Collectively, these data suggest that the circSNHG5/miR-495-3p axis utilizes CITED2 in its regulation of CEP degeneration.

## Discussion

Cartilage endplate degeneration is a well-established precursor to IDD, due to its hindrance of nutrient transport to ID (Wong et al., 2019). As such, the underlying pathways involved need to be urgently investigated. CircRNAs are ncRNAs that are ubiquitously available and are tissue-specific (Chen, 2016; Chen and Schuman, 2016; Kristensen et al., 2019). Recent evidences suggest a correlation between some circRNAs and progression of diseases (Chen et al., 2020; Gao et al., 2020; Zeng et al., 2020). However, the roles of circRNAs in the development and progression of degenerative CEP are largely unknown. As such, investigations into the underlying circRNA-mediated mechanism of CEP degradation may open up new therapeutic targets for the treatment IDD.

The present study is a novel identification of the expression patterns and functional analysis of circRNAs in degenerative versus normal CEP tissues. We detected 435 highly expressed circRNAs and 143 were downregulated circRNAs in the degenerative CEP tissues. Subsequently, among the top 30 downregulated circRNAs, we selected three circRNAs with the highest expression level for qRT-PCR verification. The results showed that circSNHG5 was remarkably decreased and abundantly expressed in degenerative CEP tissues. CircSNHG5 is located on chromosome 6q14.3 and is aligned in a sense orientation to the known long non-coding RNA (lncRNA) SNHG5. SNHG5 (also known as U50HG, gene ID: 387066) is a 524-bp lncRNA (Li et al., 2020). In prior studies, SNHG5 was reported to have low expression in osteoarthritic cartilage tissues (Shen et al., 2018), and SNHG5 silencing augmented IL-1β-stimulated apoptosis in chondrocytes (Jiang et al., 2020). In our study, we discovered a circular variation of SNHG5, which is ubiquitously expressed in human CEP tissues and was decreased expression during IDD progression. As a result, the expression pattern of circSNHG5 suggests association with IDD. To test this further, we employed silencing techniques to show that circSNHG5 participates in the cell proliferation and protection of ECM components from degradation. As such, circSNHG5 may be a potential therapeutical target in the management of IDD.

Various forms of RNAs have the capacity to serve as ceRNA during physiological and pathophysiological conditions of the cell, including circular RNAs, lncRNAs, small non-coding RNAs, pseudogenes, and mRNAs (Li et al., 2015; Sanchez-Mejias and Tay, 2015; Han et al., 2018). Additionally, multiple established ceRNA networks have been shown to contribute to disease pathogenesis and progression (Tay et al., 2014; Beermann et al., 2016; Li et al., 2018). In this paper, using bioinformatics, we discovered 12 miRNAs with sequence homology with circSNHG5. We further verified their expression in degenerative CEP tissues, relative to healthy CEP, using qRT-PCR. The miRNA with the largest fold change was miR-495-3p, which was markedly increased in degenerative CEP. Moreover, in prior studies, it was shown that miR-495-3p silencing inhibits chondrocyte apoptosis while leading to enhanced chondrocyte viability in an osteoarthritic model (Yang et al., 2019; Zhao et al., 2019). Using gain-of-function research, we confirmed a similar role of miR-495-3p in chondrocytes. Next, we established direct interaction between circSNHG5 and miR-495-3p and confirmed the interaction using luciferase and FISH analyses. Lastly, we performed rescue experiments to show that miR-495-3p silencing can successfully reverse the effects of circSNHG5 silencing in C28/I2 cells. Taken together, these results indicate that circSNHG5 sequesters miR-495-3p activity in the degenerative CEP.

Next, using bioinformatics analysis, we identified potential mRNA targets of miR-495-3p, among which, CITED2 was found to be most strongly suppressed in degenerative CEP tissues. CITED2 (CBP/p300-interacting transactivator with ED-rich tail 2), is a transcriptional regulator that negatively regulates MMP *via* the p300–Ets-1 pathway (Yokota et al., 2003; Sun, 2010). In a prior study, CITED2 was shown to mitigate cartilage degradation in osteoarthritic mice (He et al., 2019). In this study, CITED2 expression was found to be remarkably low in miR-495-3p-overexpressed cells, which is similar to another published report (Clark and Naya, 2015). Lastly, CITED2-overexpressed cells reversed the cellular response seen with circSNHG5 silencing. Taken together, we propose that the circSNHG5/miR-495-3p/CITED2 axis may, in part, be responsible for CEP degeneration and targeting this pathway may be beneficial to IDD therapy.

## Conclusion

In summary, the circSNHG5/miR-495-3p/CITED2 pathway contributes to the development and progression of IDD. Based on our results, upregulating circSNHG5 and CITED2 expression may prevent ECM degradation and miR-495-3p suppression may arrest IDD progression.

## Data Availability Statement

The datasets presented in this study can be found in online repositories. The names of the repository/repositories and accession number(s) can be found in the article/Supplementary Material.

## Ethics Statement

Written informed consent was obtained from the individual(s) for the publication of any potentially identifiable images or data included in this manuscript.

## Author Contributions

JZ and XC designed the experiments. SH, RD, and JY carried out the experiments and analyzed the experimental results. JJ and TW wrote the manuscript. All authors approved the final manuscript.

## Conflict of Interest

The authors declare that the research was conducted in the absence of any commercial or financial relationships that could be construed as a potential conflict of interest.

## Footnotes

1 https://circinteractome.nia.nih.gov/

2 http://www.targetscan.org/vert_72/

3 http://mirdb.org/

4 http://www.ncbi.nlm.nih.gov/geo

5 https://circinteractome.nia.nih.gov/

## Supplementary Material

Supplementary Table 1 Selected patient characteristics.

Supplementary Table 2 Primers and sequences used in this study.
