# Supplementary material for: CircSNHG5 Sponges Mir-495-3p and Modulates CITED2 to Protect Cartilage Endplate From Degradation
Source: Front Cell Dev Biol. 2021 Jul 1;9:668715. doi: 10.3389/fcell.2021.668715 (PMC8281349; doi:10.3389/fcell.2021.668715)
Supplement: Supplementary Table 1 — Selected patient characteristics. [file Table_1.DOCX]

**Supplementary Table 1. Selected patient characteristics.**

| **Sample** | **Gender** | **Age（year）** | **Usage** |
| --- | --- | --- | --- |
| Deg 1 | Female | 81 | qRT-PCR |
| Deg 2 | Female | 75 | qRT-PCR |
| Deg 3 | Male | 68 | qRT-PCR |
| Deg 4 | Female | 64 | qRT-PCR |
| Deg 5 | Male | 61 | qRT-PCR |
| Deg 6 | Male | 60 | qRT-PCR |
| Deg 7 | Male | 50 | qRT-PCR |
| Deg 8 | Female | 49 | qRT-PCR |
| Deg 9 | Female | 70 | qRT-PCR |
| Deg 10 | Male | 64 | qRT-PCR |
| Deg 11 | Male | 61 | qRT-PCR |
| Deg 12 | Male | 50 | qRT-PCR |
| Deg 13 | Male | 40 | qRT-PCR, Western blotting |
| Deg 14 | Male | 81 | qRT-PCR, Western blotting |
| Deg 15 | Female | 49 | qRT-PCR, Western blotting |
| Deg 16 | Female | 43 | qRT-PCR, Western blotting |
| Deg 17 | Female | 51 | qRT-PCR, Western blotting |
| Deg 18 | Male | 62 | qRT-PCR, Western blotting |
| Deg 19 | Male | 57 | qRT-PCR, Western blotting |
| Deg 20 | Female | 43 | qRT-PCR, Western blotting |
| Deg 21 | Male | 61 | qRT-PCR, Western blotting |
| Normal 1 | Male | 40 | qRT-PCR |
| Normal 2 | Male | 48 | qRT-PCR |
| Normal 3 | Female | 46 | qRT-PCR |
| Normal 4 | Female | 49 | qRT-PCR |
| Normal 5 | Male | 45 | qRT-PCR |
| Normal 6 | Female | 45 | qRT-PCR |
| Normal 7 | Female | 40 | qRT-PCR |
| Normal 8 | Male | 17 | qRT-PCR |
| Normal 9 | Male | 36 | qRT-PCR |
| Normal 10 | Male | 42 | qRT-PCR |
| Normal 11 | Female | 35 | qRT-PCR |
| Normal 12 | Male | 31 | qRT-PCR |
| Normal 13 | Female | 42 | qRT-PCR, Western blotting |
| Normal 14 | Female | 31 | qRT-PCR, Western blotting |
| Normal 15 | Female | 45 | qRT-PCR, Western blotting |
| Normal 16 | Male | 36 | qRT-PCR, Western blotting |
| Normal 17 | Female | 61 | qRT-PCR, Western blotting |
| Normal 18 | Male | 58 | qRT-PCR, Western blotting |
| Normal 19 | Male | 51 | qRT-PCR, Western blotting |
| Normal 20 | Male | 55 | qRT-PCR, Western blotting |
| Normal 21 | Male | 69 | qRT-PCR, Western blotting |

Deg, degeneration group; Normal, normal group; qRT-PCR, quantitative real-time PCR.
