# Supplementary material for: CircSNHG5 Sponges Mir-495-3p and Modulates CITED2 to Protect Cartilage Endplate From Degradation
Source: Front Cell Dev Biol. 2021 Jul 1;9:668715. doi: 10.3389/fcell.2021.668715 (PMC8281349; doi:10.3389/fcell.2021.668715)
Supplement: Supplementary Table 2 — Primers and sequences used in this study. [file Table_2.DOCX]

**Supplementary Table 2. Primers and sequences used in this study.**

| Primers for qPCR | | |
| --- | --- | --- |
| CircSNHG5 | F | AAAATCCACTAAGTAAGAAGCAAGG |
|  | R | GAACGCTGTTCACTGAAGTATAAAA |
| Hsa_circ_0071102 | F | TCATTGTTCCAGCCAGTCTG |
|  | R | GCATCGTTCATCATCTGTCA |
| Hsa_circ_0077257 | F | TCGAATGTGACTGACTAGCA |
|  | R | ACACTCAGAACGCTGTTCAT |
| MMP13 | F | GGAAGACCCTCTTCTTCTCT |
|  | R | TCATAGACAGCATCTACTTTGTT |
| COL2A1 | F | TGAGGGCGCGGTAGAGACCC |
|  | R | TGCACACAGCTGCCAGCCTC |
| Aggrecan | F | CATTCACCAGTGAGGACCTCGT |
|  | R | TCACACTGCTCATAGCCTGCTTC |
| CITED2 | F | TGCCGCCCAATGTCATAG |
|  | R | CTGCTGTTTGCACACGAAG |
| GADPH | F | CAAGGTCATCCATGACAACTTTG |
|  | R | GTCCACCACCCTGTTGCTGTAG |
| U6 | F | CTCGCTTCGGCAGCACATATACT |
|  | R | ACGCTTCACGAATTTGCGTGTC |
| miR-892b | F | ACACTCCAGCTGGGCACTGGCTCCTTTCTG |
|  | R | CTCAACTGGTGTCGTGGAGTCGGCAATTCAGTTGAGTCTACCCA |
| miR-891b | F | ACACTCCAGCTGGGTGCAACTTACCTGAGT |
|  | R | TGGTGTCGTGGAGTCG |
| miR-587 | F | CCAGGCAAGAGAGAGTTGCTG |
|  | R | AGTCACAGGTGCAGACACATT |
| miR-516b | F | AGGCATCTGGAGGTAAGAAG |
|  | R | GTTGTGGTTGGTTGGTTTGT |
| miR-495-3p | F | ACACTCCAGCTGGGAAACAAACATGGTGCA |
|  | R | TGGTGTCGTGGAGTCG |
| miR-377 | F | GTCGTGGAGTCGGCAATT |
|  | R | GGCATCACACAAAGGCAAC |
| miR-155 | F | ACGCTCAGTTAATGCTAAT CGTGA |
|  | R | ATTCCATGTTGTCCACTGTCTCTG |
| miR-1324 | F | ACACTCCAGCTGGGCCAGACAGAATTCTATGC |
|  | R | CTCAACTGGTGTCGTGGAGTCGGCAATTCAGTTGAGGAAAGTGC |
| miR-1299 | F | ACACTCCAGCTGG GTTCTGGAAUUCTC |
|  | R | CTCAACTGGTGTCGTGG AGTCGGCAATTCAGTTGAGTCCCTCAC |
| miR-1290 | F | AGCGTGTGTCGTGGAGTC |
|  | R | TCGTGAGATGAAGCACTGTAG |
| miR-127-5p | F | GCCGAGCTGAAGCTCAGAGG |
|  | R | CTCAACTGGTGTCGTGGA |
| miR-1204 | F | AGCGTCGTGGCCTGGTCTC |
|  | R | ATCCAGTGCAGGGTCCGAGG |
|  |  |  |
| siRNAs sequences | | |
| siRNA-NC |  | ACGUGACACGUUCGGAGAATT |
| CircSNHG5 si-1 |  | CGCCUAAUUCUUUUCCUUGTT |
| CircSNHG5 si-2 |  | UCACGCCUAAUUCUUUUCCTT |
|  |  |  |
| miRNA sequences | | |
| MiR-495-3p inhibitor |  | RiboBio |
| Inhibitor NC |  | RiboBio |
| Mimic NC |  | RiboBio |
| MiR-495-3p mimic |  | RiboBio |
|  |  |  |
| Probes for FISH | | |
| Alexa flour 488- circSNHG5 |  | TCAGAACGCTGTTCATTAGTGGATTTTCC |
| Cy3- miR-495-3p |  | AAGAAGTGCACCATGTTTGTTT |

**CircSNHG5 sequences:**

GGCGTGAGCCACCACCCCCGGCCCACTTTTTGTAAAGGTACGTACTAATGACTTTTTTTTTATACTTCAGTGAACAGCGTTCTGAGTGTGGACGAGTAGCCAGTGAAGATAATGAATGTCGAATGTGACTGACTAGCAGCTTCATTTTGAATGAGGGTCGCTGTCTGCCCATTGATAGAGGCCAGATTGTCTTGGAAGTTCCAAAGTTGCAACGATTTCTGGCTAGTGCCACGAGGTTTACTTGACTGTTGTGTGAAAAGCTGATAAGAAAACCATCCAGAAAAAAGCTCTTCGTTTTACAAACATGAAAATAAAACATGTAATTTTGGATTATGTTCCTTTTTGTTATTACTTTTAAATAGGTCCTGAAATAACATGGGGAGCATTAAATGGAAAATCCACTAAGTAAGAAGCAAGGAAAAGAATTA
